# Current state of research on copper complexes in the treatment of breast cancer

**DOI:** 10.1515/biol-2022-0840

**Published:** 2024-03-28

**Authors:** Kui Hu, Jingna Guo, Jiemin Zeng, Yunhao Shao, Binhua Wu, Jian Mo, Guixi Mo

**Affiliations:** Department of Anesthesiology of Affiliated Hospital, The Marine Biomedical Research Institute, Guangdong Medical University, Zhanjiang, Guangdong, 524023, China; The Marine Biomedical Research Institute of Guangdong Zhanjiang, Zhanjiang, Guangdong, 524023, China; Southern Marine Science and Engineering Guangdong Laboratory (Zhanjiang), Zhanjiang, Guangdong, 524023, China

**Keywords:** copper complexes, breast cancer, cell death

## Abstract

Breast cancer, a malignancy originating from the epithelium or ductal epithelium of the breast, is not only highly prevalent in women but is also the leading cause of cancer-related deaths in women worldwide. Research has indicated that breast cancer incidence is increasing in younger women, prompting significant interest from scientists actively researching breast cancer treatment. Copper is highly accumulated in breast cancer cells, leading to the development of copper complexes that cause immunogenic cell death, apoptosis, oxidative stress, redox-mediated cell death, and autophagy by regulating the expression of key cell death proteins or assisting in the onset of cell death. However, they have not yet been applied to clinical therapy due to their solubility in physiological buffers and their different and unpredictable mechanisms of action. Herein, we review existing relevant studies, summarize the detailed mechanisms by which they exert anti-breast cancer effects, and propose a potential mechanism by which copper complexes may exert antitumor effects by causing copper death in breast cancer cells. Since copper death in breast cancer is closely related to prognosis and immune infiltration, further copper complex research may provide an opportunity to mitigate the high incidence and mortality rates associated with breast cancer.

## 1 Introduction

Copper is a vital catalyst for hematopoiesis and iron absorption and is the third most abundant trace element in the body after zinc and iron [1]. Copper transport and cellular levels are meticulously regulated due to the potential for cellular damage arising from copper deficiency and overload. Excessive copper levels can catalyze the generation of toxic reactive oxygen species (ROS), which possess profound cytotoxic properties and can induce cellular injury [2]. Extensive studies have demonstrated a correlation between copper deprivation and clinical manifestations of anemia, leukopenia, neutropenia, and allogeneic cytopenia [3]. Moreover, infants with low birth weight and young children suffering from copper deficiency often present with bone abnormalities including osteoporosis, fractures, and skeletal deformities [4]. Copper deficiency can also give rise to clinical manifestations resembling the neurological symptoms observed in spinal cord neuropathy associated with vitamin B12 deficiency [5]. Notably, copper may be a limiting factor in various aspects of tumor progression including growth, angiogenesis, and metastasis [6]. Several copper compounds have demonstrated potent antitumor and antimetastatic properties against diverse solid tumors [7,8,9]. Furthermore, the biocompatibility and reduced toxicity of copper, along with its enhanced biological availability and increased levels in cancer tissue, position it as a potential therapeutic agent in cancer treatment [10]. Breast cancer treatment involves surgery, immunotherapy, radiotherapy, and chemotherapy. However, immunotherapy is only available to certain patients, and radiotherapy and chemotherapy have a serious impact on patients’ quality of life. Consequently, copper complexes are an innovative alternative for breast cancer treatment. These complexes are stable with specific functionalities formed through the complexation of copper ions with metal nuclei in appropriate organic ligands, leading to a diverse array of copper complexes. Nonetheless, challenges persist regarding their solubility in physiological buffers, as well as their multifaceted and unpredictable mechanisms of action preventing their clinical application [11]. With ongoing research endeavors, the prospects of complexes for cancer treatment appear boundless.

Breast cancer, characterized by the malignant growth of the breast epithelial or ductal epithelial cells, is the leading cause of cancer-related mortality among women worldwide, with metastatic disease accounting for most breast cancer-related deaths [12]. Despite extensive research efforts spanning experimental, epidemiological, and clinical research over several decades, the incidence of breast cancer continues to rise [13]. Breast cancer caused 685,000 female deaths worldwide in 2020, with nearly two-thirds of these deaths in less developed regions [14], and more than half of all breast cancers occur in women under 50 years of age [15]. Breast cancer imposes a substantial burden on overall women’s health, and despite the increasing diversity of treatment options available, the persistently high rates of morbidity and mortality remain unchanged.

Immunogenic cell death (ICD) is a functionally unique form of stress-driven regulatory cell death, characterized by its capacity to elicit an inflammatory cellular response. ICD mechanisms include exposure to microbe-associated molecular patterns (MAMPs) or damage-associated molecular patterns (DAMPs) and endoplasmic reticulum (ER) chaperone proteins. MAMP interaction with pattern recognition receptors (PRRs) creates ideal conditions for the initiation of antigen-specific immune responses [16]. PRRs can also be activated by DAMP, and PRR signaling can activate endogenous adaptive immunity [17], artificially increasing the availability of specific DAMP to induce ICD [18]. Additionally, eIF2A phosphorylation-dependent exposure to ER chaperone proteins can cause ICD, such as calreticulin (CALR) [19], protein disulfide bond isomerase A family member 3 (PDIA3; also known as ERp57) [20], heat-shock protein 70 kDa (HSP70; also known as HSPA1A35), and heat-shock protein 90 kDa (HSP90; also known as HSP90AA1) [21]. This development of adaptive immunity in T-cells can in turn kill tumor cells.

Apoptosis is a fundamental biological process essential for maintaining tissue homeostasis and eliminating damaged cells in both physiological and pathological conditions. It is highly regulated and plays a vital role in various aspects of growth and development [22]. The two primary pathways involved are the extrinsic or death receptor pathway and the intrinsic or mitochondrial pathway [23]. Additionally, there is an alternative pathway involving T cell-mediated cytotoxicity and perforin granzyme-dependent cell killing. Of note, the extrinsic, intrinsic, and granzyme B pathways share a common pathway initiated by caspase-3 cleavage, leading to DNA fragmentation, degradation of cytoskeletal and nuclear proteins, protein cross-linking, formation of apoptotic vesicles, expression of phagocyte receptor ligands, and, finally, uptake by phagocytes. In contrast, the granzyme A pathway is a parallel, cysteine-independent signaling cell death pathway that can be stimulated by single-stranded DNA damage [24]. Cysteases cleave proteins at aspartate residues [25], and several major cysteases have been identified and broadly classified as promoters (caspase-2, -8, -9, -10), effectors or executors (caspase-3, -6, -7), and inflammatory cystathiases (caspase-1, -4, -5) [26,27].

Oxidative stress arises from the disrupted redox homeostasis maintained by cell aerobic metabolism [28] and is characterized by excess ROS production [29]. ROS are generated as byproducts during normal cell metabolism, but prolonged high ROS levels can extensively damage cellular structures and functions, resulting in both somatic mutations and the transformation of normal cells into cancerous cells [22,30], as well as oxidative damage leading to the overexpression of oncogenes and other molecular alterations, ultimately to tumorigenesis [31]. Tumorigenesis involves DNA mutations, DNA damage, genomic instability, and cell expansion, collectively facilitating cancer initiation and progression [32].

Autophagy is a cellular mechanism that protects and eliminates stressed cells [33], as well as degrades proteins and organelles [33,34]. There are three forms: macroautophagy, microautophagy, and selective autophagy [35]. Autophagy plays a crucial role in the adaptive and innate immune systems, mainly through its ability to eliminate intracellular pathogens, deliver antigens to the MHC class II restriction zone, and facilitate the transport of viral nucleic acid to toll-like receptors [33,36]. Autophagy regulation involves ULK1, class III phosphoinositide 3-kinase composed of five subunits (ATG14L, Beclin 1, VSP34, and VSP15), ATG5, ATG12, ATG16L, and LC3II (lipidated microtubule-associated protein light chain 3).

**Figure 1:**
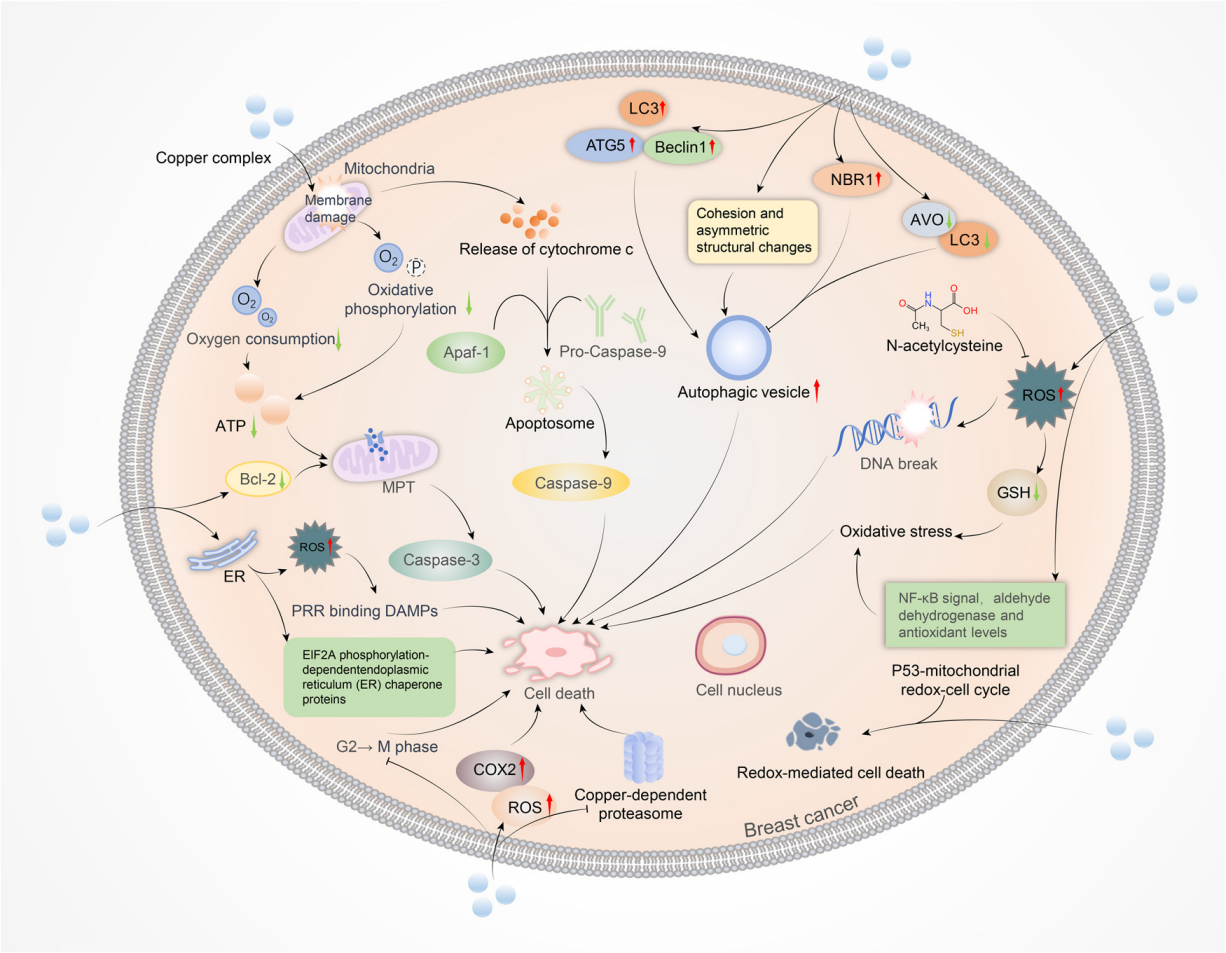
Copper complexes exert anti-breast cancer mechanisms. ICD, apoptosis, oxidative stress, redox-mediated cell death, and autophagy are involved in the anti-breast cancer processes.

Copper death is a new type of cell death first proposed by Tsvetkov et al. and involves copper binding to the sulfurylated components of the tricarboxylic acid cycle. This process leads to the aggregation of sulfurylated proteins and subsequent loss of iron–sulfur cluster proteins, resulting in proteotoxic stress and ultimately cell death [37]. Elevated copper levels have been observed in various cancers, such as breast, prostate, lung, and brain cancers [38,39,40,41,42]; therefore, it may be a potential tumor-specific target for anti-cancer therapies [42,43]. Several researchers are investigating the potential of copper complexes, which involve the complexation of copper with specific organic compounds. These copper complexes induce various modes of cell death, including ICD, apoptosis, ROS-induced cell death, and autophagy (Figure 1). These modes of cell death are considered the primary mechanisms of action for copper compounds. This article elucidates the anti-breast cancer mechanisms of copper complexes, proposing a potential mechanism by which copper complexes exert antitumor effects by inducing copper death in breast cancer cells.

## 2 Mechanism of action of copper complexes in breast cancer therapy

### 2.1 Copper complexes can cause ICD in breast cancer cells

ICD is a type of regulatory cell death capable of eliciting an inflammatory cellular response that enables the recognition of tumor-associated antigens by T cells, thereby promoting an immune response against the tumor. Cancer stem cells (CSCs) are a subpopulation of cancer cells that can self-renew, differentiate, and form secondary tumors [44]. They can also evade conventional chemotherapy and radiotherapy because of their stem cell-like growth characteristics [45]. They can regenerate the original tumor and/or generate invasive cancer cells that can colonize distant organs [46]. Thus, CSCs are potential therapeutic targets, and their death may become the ultimate approach to eradicating breast cancer. Schiff base compounds are organic ligands that readily form complexes with metal ions. Schiff base metal complexes are structurally rich, stable, and exhibit good catalytic properties and biological activities. In a groundbreaking study by Kaur et al., a novel copper complex incorporating a Schiff base ligand and a polypyridine [47] was demonstrated to induce ICD specifically in mammary stem cells and enhance phagocytosis by immune cells. Its mechanism of action involves the polypyridyl ligand 4 entering the ER of CSCs, thus increasing intracellular ROS levels. Inducing ER stress may cause eIF2A phosphorylation-dependent exposure of ER chaperone proteins, inducing DAMP binding to PRRs to cause ICD, which in turn exerts a therapeutic effect on breast cancer.

### 2.2 Copper complexes exert therapeutic effects in breast cancer through apoptosis

Excessive apoptosis can contribute to degenerative diseases, while inadequate apoptosis is implicated in cancer development [48]. Pistritto et al. highlighted that the hallmark of cancer is disturbed apoptotic cell death mechanisms and that dysregulated apoptosis is associated with tumor initiation and progression, as well as tumor resistance to therapy [49]. Hanahan et al. also proposed that dysregulated apoptosis is a characteristic of cancer [50]. The crucial determinant of apoptosis is the equilibrium between pro- and anti-apoptotic proteins [42], but apoptosis can be regarded as an edged sword, with the capacity to act as a catalyst and a resolution for cancer progression [51,52]. Consequently, numerous investigators have endeavored to induce cancer cell apoptosis to achieve therapeutic effects. Therapeutic strategies involving apoptotic pathway activation or the elimination of apoptosis inhibitors in cancer cells have proven promising approaches for cancer management [53]. Hence, new drugs targeting various aspects of apoptosis in cancer cells are being developed [54,55]. Cui et al. [56] designed a new low-toxicity copper depletion nanoparticle (CDN) that can cause apoptosis in triple-negative breast cancer cells (TNBCs) by reducing oxygen consumption and oxidative phosphorylation, decreasing ATP production coupled with impaired mitochondrial membrane potential and elevated oxidative stress. Foo et al. [57] also developed a new copper complex [Cu(SBCM)2] that causes apoptosis in TNBCs by downregulating Bcl-2 expression to release cytochrome C in mitochondria. The compounds developed by Cui et al. [56] and Foo et al. [57] cause alterations in mitochondrial membrane permeability. As demonstrated by Cui et al., CDN that specifically targets mitochondria can effectively decrease ATP production in TNBCs by reducing oxygen consumption and oxidative phosphorylation, consequently prompting a metabolic shift toward glycolysis as the primary energy source. This energy depletion, coupled with the impaired mitochondrial membrane potential and increased oxidative stress, ultimately culminates in the activation of the mitochondrial permeability transition pore to release multiple pro-death factors in the mitochondrial membrane interstitial space into the cytoplasm, triggering caspase-3 activation and apoptosis [56]. The copper complex [Cu (SBCM)2] developed by Jhi Biau Foo et al. induced cytochrome c release, which, in turn, activates Apaf-1 and procaspase-9 to form an “apoptosome,” thereby downregulating anti-apoptotic protein Bcl-2 in breast cancer cells, followed by a change in cell membrane permeability [58,59,60].

In addition, several other copper complexes have been developed with potential anti-cancer properties, including the ternary copper complex (1,10-phenanthroline and tyrosine [Cu(phen)(L-tyr)Cl]⸱3H_2_O) [61], five ternary copper(ii) complexes [Cu(OH-PIP)(Phe)Cl (1), Cu(OH-PIP)(Gly)(H_2_O), NO_3_-2H_2_O (2), Cu(OH-PIP)(Ala)(Cl)-H_2_O (3), Cu(OH-PIP)(Met)PF_6_-2H_2_O (4), and Cu(OH-PIP)(Gln)(H_2_O)(Cl)-3H_2_O (5)] [62], and copper(i)–nicotinate complex (CNC) [63]. Their mode of action involves the direct or indirect regulation of anti-apoptotic (such as Bcl-2 and Bcl-*x*) and pro-apoptotic (such as Bcl-10 and Bax) proteins, leading to the expression of caspase-9, caspase-3, and caspase-7, key players in apoptosis to induce the apoptosis of breast cancer cells. Jimin Shin et al. developed a biostable copper(ii)–NSAID complex to induce apoptosis in CSCs through the activation of the ROS [64] and cyclooxygenase isozyme 2-dependent apoptotic pathway [65]. Furthermore, Morais et al. synthesized six phosphane Cu(i) complexes with N, N, N, O, and N, S bidentate ligands, which exhibit remarkable antitumor cytotoxicity, primarily achieved through the inhibition of the G2/M cell cycle and apoptosis in breast cancer cells [66].

In addition to promoting the expression of apoptotic genes or inhibiting apoptosis-inhibiting enzymes, there is evidence that inhibiting the copper-dependent proteasome may also induce apoptosis in breast cancer cells. This finding holds potential therapeutic implications for breast cancer treatment. Meanwhile, various derivatives of 8-hydroxyquinoline and their copper complexes exhibited selective suppressive effects on the growth of MDA-MB-231 breast cancer cells. They could also induce cancer cell apoptosis by interfering with the activation of the copper-dependent proteasome [42].

The CDNs with diverse chemical structures possess therapeutic potential, inducing apoptosis in breast cancer cells through the extrinsic or death receptor pathway and the intrinsic or mitochondrial pathway; therefore, the induction of apoptosis by copper complexes holds promise as a potential strategy to mitigate the high morbidity and mortality rates in breast cancer patients.

### 2.3 Copper complexes induce oxidative stress and redox-mediated cell death in breast cancer cells

Oxidative stress in the context of breast cancer can have a dual nature, acting as a double-edged sword, contributing to breast cancer development and progression as well as holding therapeutic potential for breast cancer treatment. Copper complexes possess cytotoxic properties, inducing oxidative stress in breast cancer cells, which in turn might have a therapeutic impact on breast cancer.

Copper ferrite (CuFe_2_O_4_) nanoparticles (NPs) [67] are a significant spinel ferrite due to the phase transitions, semiconducting property changes, electrical switching, and tetragonality variation under different conditions. The isoflavone dye lignin [68] is a plant polyphenol believed to have chemopreventive and therapeutic properties. Johnson et al. synthesized a copper(i) complex comprising non-steroidal anti-inflammatory drugs (NSAIDs) and triphenylphosphine ligands (1–3) [69]. The complex was internalized by breast CSCs, increasing intracellular ROS levels and depleting glutathione leading to cell death. Furthermore, these copper complexes induced cell death in breast cancer cells, and this effect was counteracted by the addition of *N*-acetylcysteine, a ROS scavenger. Similarly, Zubair et al. revealed that copper-containing substances, specifically the antioxidant citronellol and its semisynthetic derivative apocryptosterone [70], induced excessive ROS generation in breast cancer cells, subsequently causing ROS-mediated cellular DNA breaks and cell death. In addition, Allensworth et al. also elucidated that copper-containing substances, specifically DSF complexed with copper (DSF-Cu), induced cell death corresponding to XIAP and eIF4G1 downregulation, and initiated oxidative stress-mediated apoptosis in breast cancer cells by modulating NFκB signaling, aldehyde dehydrogenase activity, and antioxidant levels. Consequently, these molecular events culminate in the manifestation of antitumor efficacy in inflammatory breast cancer [71].

Copper ligands possess therapeutic potential beyond their ability to induce oxidative stress in breast cancer cells. Periasamy et al. found that the ligand copper(ii) compound [Cu(tdp)(phen)](ClO_4_) exhibited a high binding affinity to p53-mitochondrial redox-cell cycle coupling. Consequently, this interaction led to the activation of redox-mediated cell death pathways, specifically within breast cancer cells. These findings contribute to our understanding of the intricate molecular mechanisms by which copper ligands can elicit therapeutic effects in the context of breast cancer [72].

Oxidative stress can cause mutations in intracellular genomic DNA to promote tumorigenesis and progression, as well as play a therapeutic role in tumor therapy by regulating the expression of specific genes. These copper-containing compounds selectively induce oxidative stress in tumor cells while sparing normal cells. The continuous improvement of the biocompatibility and targeting ability of copper complexes, as well as the continuous reduction of their toxicity, means that copper-containing complexes have great application prospects for breast cancer treatment.

### 2.4 Copper complexes may exert therapeutic effects in breast cancer through autophagy

P62, despite its multi-domain nature within signal transduction junctions, assumes a pivotal role as a key determinant in cell death and survival [73,74]. In breast cancer cells, P62 exerts a bridging function in autophagy regulation, mainly attributed to its interaction with ubiquitinated proteins that are earmarked for degradation. Furthermore, P62 also forms a complex with LC3II during autophagic vesicle formation, resulting in the engulfment of targeted proteins and organelles within these autophagic vesicles, which later fuse with lysosomes facilitating the degradation of their contents [33,75]. P62 is mainly involved in breast cancer progression and metastasis [76,77]. It does not directly regulate autophagy but assists autophagy in phagocytosis and digestion. Impaired autophagy has been implicated in cancer development [78]. Consequently, efforts have been made to develop a copper complex to promote autophagy in breast cancer cells. Koňariková et al. investigated the impact of Schiff base Cu(ii) complexes on human breast cancer cells (MCF-7), demonstrating that these complexes caused cohesion and asymmetric structural changes in actin filaments. Additionally, they observed a concomitant upregulation of LC3 protein expression, subsequently resulting in the production of autophagic vesicles and the induction of autophagy. Furthermore, these Cu(ii) complexes exhibited anti-proliferative and anti-cancer effects specifically targeting MCF-7 cells [79]. Similarly, Li et al. synthesized a ternary copper compound composed of 1,10-phenanthroline and tyrosine [Cu(phen)(L-tyr)Cl]⸱3H_2_O. Notably, the presence of 3H_2_O induced LC3II expression in two breast cancer cell lines, MCF-7 and MDA-MB-231, subsequently stimulating the formation of phagocytic clusters, thereby eliciting autophagy [80]. Furthermore, Laha et al. demonstrated that transition metal copper oxide NPs (CuO NPs) are potent autophagy inducers. This copper complex upregulated LC3II, beclin1, and ATG5 protein expression, promoting the elongation of phagocytic clusters and the generation of numerous autophagic vesicles. Ultimately, this cascade of events culminated in autophagy induction in the MCF-7 cell line [81]. Abdel-Mohsen et al. demonstrated the efficacy of a CNC as a novel therapeutic agent with antitumor activity and found that it inhibited the growth of the human HCC1806 breast cancer cell line, leading to cell death in a dose-dependent manner. The observed decrease in AVO and LC3 protein levels, along with the concurrent increase in NBR1 gene expression in CNC-treated cells, provided evidence of autophagy inhibition [82]. Notably, the copper complex effectively suppressed autophagy in breast cancer cells while inducing cell death, thus highlighting its therapeutic potential in breast cancer treatment.

In summary, these newly developed copper complexes can upregulate intracellular LC3 protein. Although some studies did not specify the specific changes in LC3I and LC3II, the changes are suggestive of autophagy. LC3II is a cytoplasmic form of LC3 (LC3-I), which forms LC3-II with phosphatidylethanolamine after autophagy; therefore, LC3 expression is a reliable marker of autophagy and autophagy-related processes (including autophagic cell death).

## 3 Summary and prospects

Copper levels are in various tumors including breast cancer [38,39,40,41,42]. Since copper is inherently biocompatible and less toxic, its bioavailability and increased content in cancerous tissues make it an interesting candidate for cancer treatment. Consequently, numerous copper complexes have been developed to specifically target breast cancer. These complexes exhibit several mechanisms of action, inducing ICD, apoptosis, oxidative stress, redox-mediated cell death, and autophagy. Indeed, copper complexes may become the preferred drugs for treating breast cancer in the future due to their ability to exert anti-breast cancer effects through various mechanisms. Currently, the biocompatibility of copper complexes remains low, and their mechanisms of action and toxicity to organisms are still unpredictable; thus, they do not meet the criteria for clinical use. Therefore, further optimization and in-depth study of the detailed mechanisms of copper complexes are necessary. To date, many new copper complexes have been developed. Although their main mechanisms have been elucidated, their specific combined mechanism remains unclear, such as the six phosphane Cu(i) complexes with N, N, N, O, and N, S bidentate ligands synthesized by Morais et al. [67]. While the authors reported that these complexes stimulate apoptosis in breast cancer cells, they did not thoroughly investigate the specific pathways through which these copper complexes activate or inhibit apoptotic genes. Therefore, further studies are necessary to explore the modulation of copper compounds through more specific and detailed signaling pathways.

Ongoing research efforts aim to develop new copper complexes with improved efficacy in targeting breast cancer and minimized side effects compared to other drugs. Copper levels are increased in breast cancer cells, and this accumulation of intracellular copper causes a unique form of cell death, known as copper death [37]. However, there are still unanswered questions regarding the specific mechanism and why high copper levels may not effectively kill breast cancer cells. One possibility is that breast cancer cells have developed immunity or resistance to high copper levels, or they may counteract the effects of elevated copper by expressing specific proteins or actively excluding intracellular copper. It is also plausible that breast cancer cells have undergone adaptive mutations in response to increased copper levels. Further investigation is required to explore the exact mechanism involved. An in-depth exploration of the mechanism by which high levels of copper do not cause cell death will provide a deeper understanding of breast cancer’s mechanism and a target for other therapeutic modalities. It will also be crucial for the subsequent development of relevant copper complexes that exert antitumor effects. Notably, no previous reports have explored the ability of these copper complexes to induce breast cancer cell death by causing cellular copper overload, resulting in mitochondrial lipocalin clustering and destabilization of iron-sulfur protein clusters [37]. However, studies have identified an antitumor effect of zinc pyrithione by inducing copper death in TNBCs [83]. Interestingly, Allensworth et al. demonstrated that a copper-containing compound (disulfiram [DSF] in complex with copper [DSF-Cu]) increased cellular copper levels both in vitro and in vivo, bypassing the participation of membrane transport proteins in this process [71]. However, whether this copper complex induces copper death in breast cancer cells has not been explored. Additionally, if copper death does occur, the precise mode of action needs further investigation. This raises the question of whether all copper complexes can increase intracellular copper levels or if specific structural features enable them to do so. If newly developed or existing copper complexes incorporate compounds such as DSF or DSF-Cu, would it be relevant to determine their ability to increase intracellular copper levels in breast cancer cells? All these findings provide novel insights for targeting breast cancer cells through copper death using copper complexes. A complex of DSF and copper increases lipid peroxidation in breast cancer cells, leading to a dramatic increase in HMOX1 activity, which in turn causes cells to undergo iron death, but the researchers have not further explored whether this compound causes cells to undergo copper death [84]. Furthermore, copper complexes can also have therapeutic effects on breast cancer through the non-copper death pathway, which is more selective for the mode of action of the newly developed copper compounds and enables the development of copper compounds that can target breast cancer cells more precisely.

In conclusion, copper complexes can obstruct breast cancer cells by targeting cell death-related proteins, leading to ICD, apoptosis, oxidative stress, and autophagy. The synthesis of novel copper complexes with increased specificity means that breast cancer could be effectively treated in the coming years.
